# Compressive neuropathy of the first branch of the lateral plantar nerve: a study by magnetic resonance imaging*

**DOI:** 10.1590/0100-3984.2013.0028

**Published:** 2015

**Authors:** Rogéria Nobre Rodrigues, Alexia Abuhid Lopes, Jardélio Mendes Torres, Marina Franco Mundim, Lênio Lúcio Gavio Silva, Breno Rabelo de Carvalho e Silva

**Affiliations:** 1 MDs, Musculoskeletal Radiologists, Axial Medicina Diagnóstica, Belo Horizonte, MG, Brazil.; 2 MD, Orthopedist, Head of the Foot Surgery Group at Santa Casa de Belo Horizonte, Professor at Faculdade de Ciências Médicas de Minas Gerais (FCMMG), Belo Horizonte, MG, Brazil.; 3 MDs, Radiologists, Axial Medicina Diagnóstica, Belo Horizonte, MG, Brazil.; 4 MD, Physician Assistant, Axial Medicina Diagnóstica, Belo Horizonte, MG, Brazil.

**Keywords:** Abductor digiti quinti muscle, Baxter, Lateral plantar nerve, Inferior calcaneal nerve, Atrophy

## Abstract

**Objective:**

To assess the prevalence of isolated findings of abnormalities leading to entrapment of the lateral plantar nerve and respective branches in patients complaining of chronic heel pain, whose magnetic resonance imaging exams have showed complete selective fatty atrophy of the abductor digiti quinti muscle.

**Materials and Methods:**

Retrospective, analytical, and cross-sectional study. The authors selected magnetic resonance imaging of hindfoot of 90 patients with grade IV abductor digiti quinti muscle atrophy according to Goutallier and Bernageau classification. Patients presenting with minor degrees of fatty muscle degeneration (below grade IV) and those who had been operated on for nerve decompression were excluded.

**Results:**

A female prevalence (78.8%) was observed, and a strong correlation was found between fatty muscle atrophy and plantar fasciitis in 21.2%, and ankle varices, in 16.8% of the patients.

**Conclusion:**

Fatty atrophy of the abductor digiti quinti muscle is strongly associated with neuropathic alterations of the first branch of the lateral plantar nerve. The present study showed a significant association between plantar fasciitis and ankle varices with grade IV atrophy of the abductor digiti quinti muscle.

## Abstract (pt)

**Objetivo:**

Avaliar a prevalência de achados isolados que causam compressão do primeiro ramo do nervo plantar lateral em pacientes com queixa de dor crônica no calcanhar, cujos exames de ressonância magnética mostraram atrofia gordurosa seletiva completa do músculo abdutor do quinto dedo.

**Materiais e Métodos:**

Estudo retrospectivo, analítico e transversal. Selecionamos exames de ressonância magnética do retropé de 90 pacientes que apresentavam atrofia muscular grau IV do abdutor do quinto dedo utilizando a classificação de Goutallier e Bernageau. Foram excluídos do estudo pacientes com níveis menores de degeneração muscular (abaixo do grau IV).

**Resultados:**

Houve predomínio do sexo feminino de 78,8% e alto índice de concordância da atrofia gordurosa do músculo abdutor do quinto dedo com fasciite plantar e varizes no tornozelo, respectivamente, encontrados em 21,2% e 16,8% dos pacientes.

**Conclusão:**

Atrofia gordurosa do músculo abdutor do quinto dedo está fortemente associada a alterações neuropáticas do primeiro ramo do plantar lateral. Nosso estudo mostrou associação significativa entre a fasciite plantar e varizes do tornozelo com atrofia grau IV do abdutor do quinto dedo.

## INTRODUCTION

Heel pain is a very common complaint in orthopedic offices. The Brazilian radiological literature has recently been concerned with the relevant role played by imaging methods in the improvement of the diagnosis in the musculoskeletal system^(1-15)^.

Thalamic pain presents a wide spectrum of differential diagnosis including plantar fasciitis, fat pad involvement, stress fracture, enthesopathy and inflammatory arthropathy^(16)^. One of the most common causes of chronic pain is entrapment of the first branch of the lateral plantar nerve, a condition that is known as Baxter's neuropathy^(17,18)^. It is believed that approximately 20% of cases of pain in the medial region of the heel are associated with neuropathy of that nerve^(19-22)^.

**Figure 1:**
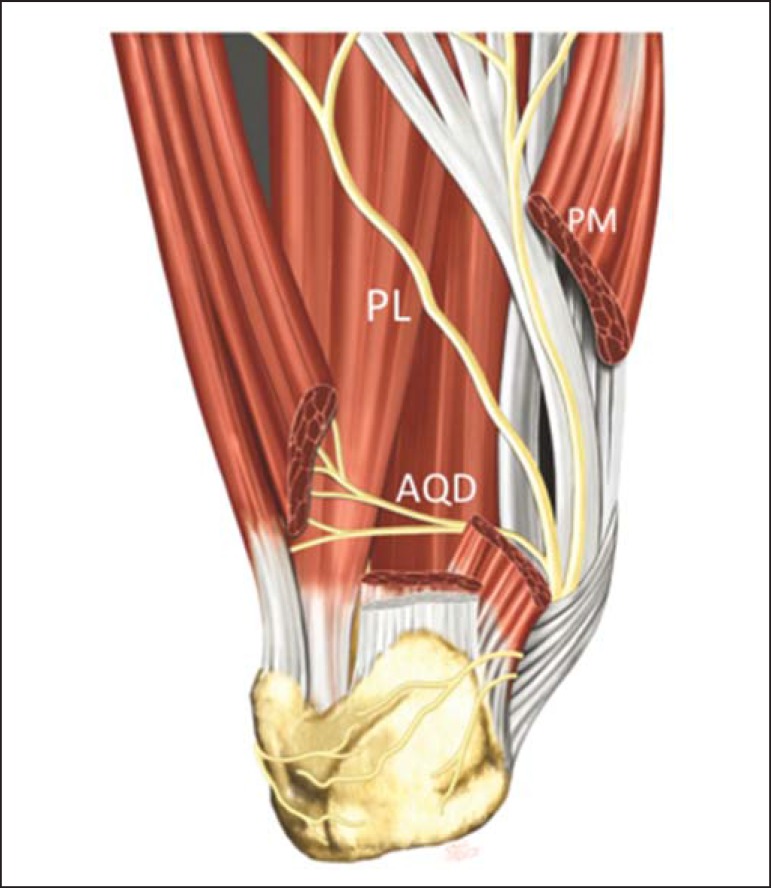
PM, medial plantar nerve; PL, lateral plantar nerve; AQD, abductor digiti quinti nerve.

Generally, the nerve to the abductor digiti quinti (ADQ) muscle originates as first branch of the lateral plantar nerve (82.1%) that divides itself at the level of the medial malleolus, but presents with some anatomical variations. In 11.7% of cases, such nerve may present as a direct branch from the posterior tibial nerve, or even originate from a common branch with the posterior branch to the lateral plantar nerve and with the medial calcaneal branch (4.1%) or in a branch in common with the posterior branch to the plantar square (2.1%)^(23)^. The nerve follows the medial pathway along the long plantar ligament to the lateral, between the abductor hallucis muscle and the medial calcaneal tuberosity, inserting into the proximal aspect of the ADQ muscle (Figure 1).

The nerve to the ADQ is mixed and originates motor nerves to the ADQ and, occasionally, to the short flexor of the digits and plantar square muscle, as well as sensory branches to the calcaneal periosteum, long plantar ligament and adjacent skin^(24)^. Any situation determining increased volume in the region of the nerve might cause a focal compressive effect with consequential neuropathy.

At clinical evaluation, the symptoms may not be distinguished from plantar fasciitis and, frequently, both conditions overlap^(25)^. ADQ muscle weakness may be present in chronic cases, with decreased fifth toe abduction strength determined by the muscle degeneration.

Magnetic resonance imaging (MRI) may be used to detect alterations associated with ADQ muscle denervation^(18,26)^. The presence of such a muscle atrophy observed at MRI reflects a chronic compression of the inferior calcaneal nerve and contributes to the clinical diagnosis of Baxter's neuropathy^(19)^.

**Figure 2:**
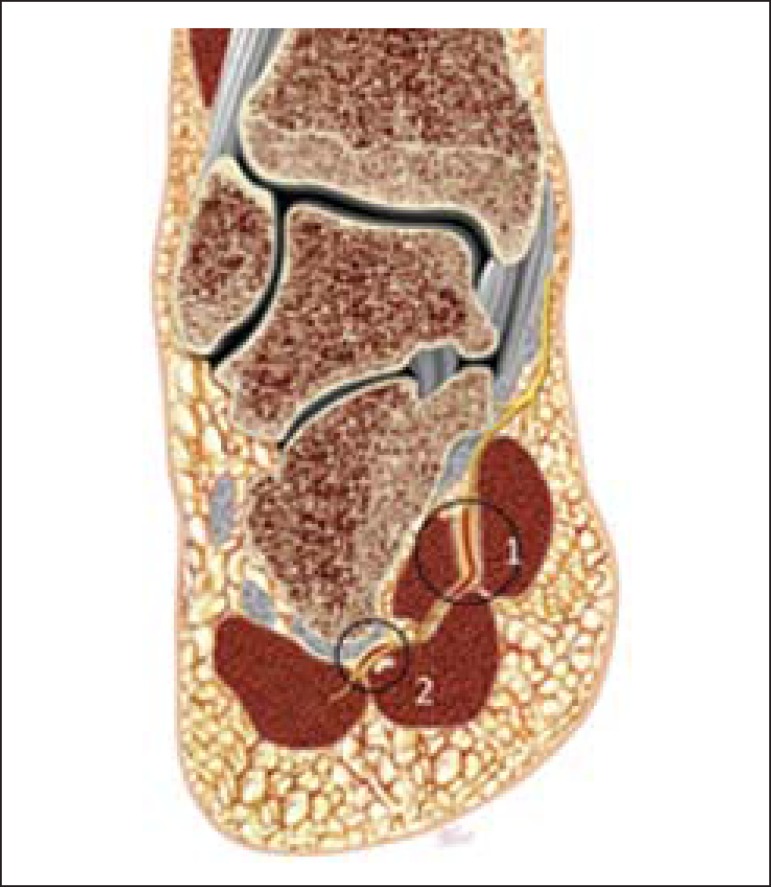
Two possible sites of nerve entrapment: 1 - in the nerve pathway between the deep fascia of the abductor muscle of the hallux and the medial plantar margin of the plantar square muscle; 2 - distally, in the nerve pathway along the medial calcaneal tuberosity.

The literature suggests two possible sites of nerve entrapment which could result in Baxter's neuropathy, as follows: the first one, in patients with altered biomechanics, such as excessive pronation, since the nerve may be compressed in the movement of lateral rotation between the plantar square and abductor hallucis muscles^(24)^; and second, either the nerve may be compressed as it passes anteriorly to the medial calcaneal tuberosity, or interfere mechanically with the plantar calcaneal spur^(17,25,27,28)^ (Figure 2).

The present study is aimed at evaluating the prevalence of MRI findings associated with compression of the first branch of the lateral plantar nerve in patients with chronic heel pain manifesting by complete selective atrophy of the ADQ muscle.

## MATERIALS AND METHODS

Retrospective, analytical, and cross-sectional study of 90 patients with diagnosis of. grade IV atrophy of ADQ muscle (according to Goutallier and Bernageau classification^(29)^) with mean age of 49.2 years, submitted to hindfoot MRI in a high field 1.5 T apparatus with fast spin echo, sagittalT1-weighted sequences and proton density (PD) with fat suppression, and axial and coronal/oblique PD T2-weighted sequences with fat suppression. After intravenous paramagnetic contrast injection, coronal and sagittal T1-weighted sequences with fat suppression were acquired. Only patients classified with grade IV atrophy of ADQ muscle (complete fatty muscle atrophy) were considered for evaluation. Amongst the evaluated patients, 21.2% were men with mean age of 42.1 years, and 78.8% were women with mean age of 56.3 years.

## RESULTS

**Figure 3:**
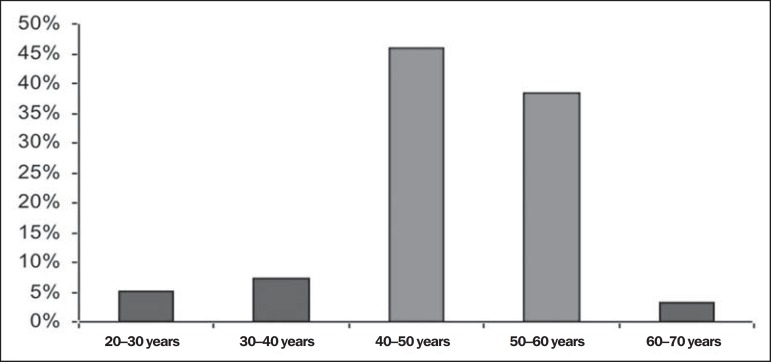
High prevalence of abductor digit quinti atrophy in patients aged between 40 and 50 years (45.9%) and between 50 and 60 years (38.3%).

**Figure 4:**
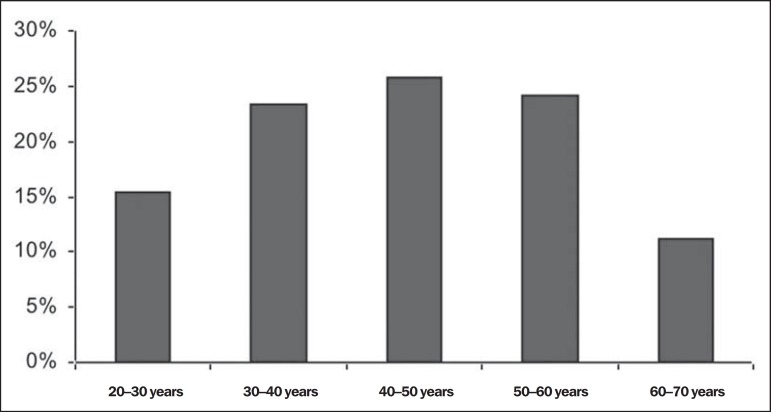
No statistically significant difference was observed as regards prevalence of ADQ muscle atrophy in male individuals.

Atrophy of the ADQ muscle was most prevalent in women - 71 cases (78.8%) -, with a high prevalence in this group at the age ranges from 40 to 50 years (45.9%) and 50-60 years (38.3%), and *p* < 0.01 in both groups as demonstrated on Figure 3. On the other hand, no statistically significant difference was observed in the prevalence of atrophy of the ADQ muscle, as demonstrated on Figure 4.

A strong correlation (*p* < 0.01) between grade IV atrophy of the ADQ muscle and plantar fasciitis and hindfoot varicosities in 21.2% and 16.8% of patients, respectively, considering isolated factors determining neural compression (Table 1).

**Table 1:** Frequency of isolated MRI findings associated with ADQ atrophy.

	Percentage	*p*-value
Plantar fasciitis	21.2%	*p* < 0.01
Varicosities	16.8%	*p* < 0.01
Lateral ligament injury	9.2%	*p* > 0.01
Medial ligament injury	1.8%	*p* > 0.01
Tendinopathy	8.4%	*p* > 0.01
Previous trauma	7.2%	*p* > 0.01
Tarsal tunnel syndrome	7.2%	*p* > 0.01
Plantar lipoma	0.9%	*p* > 0.01

In the female group, plantar fasciitis and ankle varicosities were determinant as statistically significant (*p* < 0.01), while in the male group only the trauma factor was apparently reliable as an isolated factor of compressive neural injury (Table 2).

**Table 2:** Frequency of MRI findings related to ADQ atrophy according to sex.

	Male	Female	*p*-value
Plantar fasciitis	11.5%	37.8%	*p* < 0.01
Varicosities	9.7%	31.9%	*p* < 0.01
Trauma	19.5%	11.2%	*p* < 0.01
Tendinopathy	6.5%	9.6%	*p* < 0.01
Plantar lipoma	0%	1.9%	*p* < 0.01

## DISCUSSION

MRI has shown to be an invaluable investigative method to detect muscle alterations associated with denervation. It is the most sensitive method to detect involvement of muscle tissues as compared with ultrasonography and computed tomography. Because of its noninvasive nature and capacity to demonstrate anatomical details, this method presents some advantages as compared with electromyography^(30)^.

**Figure 5:**
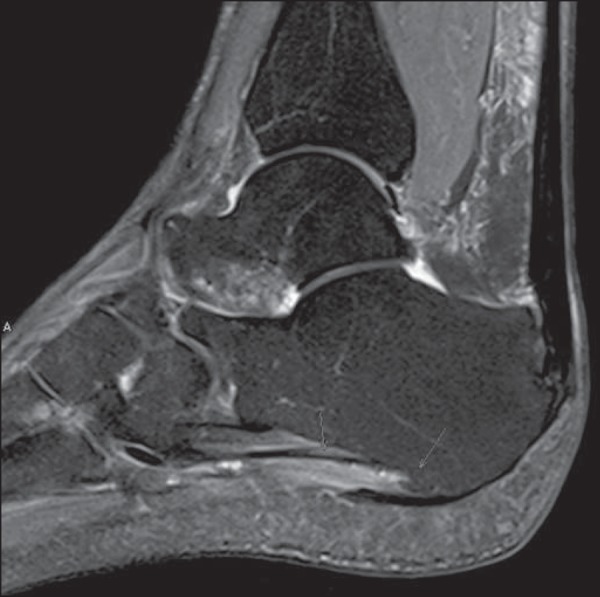
Sagittal MRI DP-weighted sequence with fat suppression showing signs of plantar fasciitis and abnormality in the signal of ADQ muscle fibers, that is hyperintense, corresponding to a pattern of edema resulting from acute denervation.

**Figure 6:**
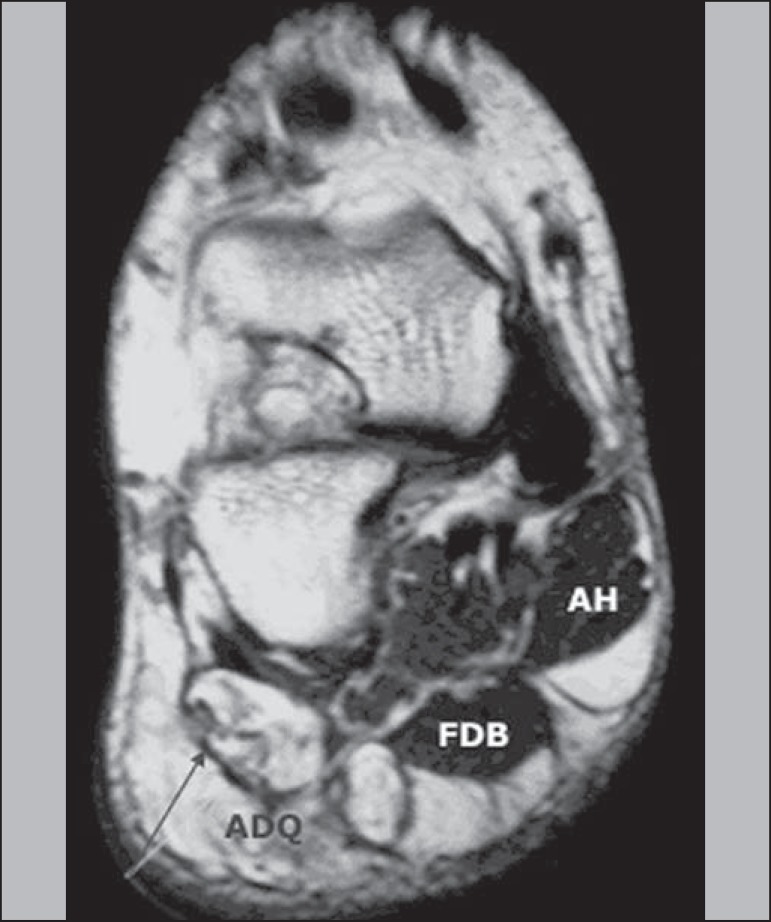
Coronal MRI T2-weighted image without fat saturation showing significant volumetric reduction of the ADQ muscle, with complete fatty infiltration of its fibers (grade IV) secondary to chronic denervation (> 1 year).

Acute and subacute muscle denervation is more appropriately evaluated at fluid-sensitive MRI sequences such as PD/T2-weighted sequences with fat suppression or STIR sequences, showing increased signal intensity within the muscle belly as compared with the normal muscle, corresponding to neurogenic muscle edema^(30,31)^ (Figure 5). The enhancement of the muscle by gadolinium also occurs either in the acute or subacute phase of denervation^(30)^. In compressive Baxter's neuropathy, muscle edema occurs selectively inside the ADQ muscle and potentially in the short flexor muscle of the toes and plantar square muscle, depending on the anatomical variation in the patient. Chronic denervation leads to muscle atrophy and subsequent irreversible fat infiltration. Such findings are clearly depicted at T1-weighted images without fat suppression^(30,31)^ (Figure 6). Typically, atrophy and fat infiltration occur homogeneously in the muscle belly. On the other hand, in the presence of double or redundant innervation, such changes either may not occur or occur heterogeneously^(30)^.

It is estimated that in 20% of patients with chronic hell pain such condition is related to compression of the abductor digiti quinti nerve^(32)^. In a study evaluating the association between ADQ atrophy and MRI findings of potential causes, there was a strong correlation between muscle atrophy and plantar fasciitis and calcaneal spur. However, the patients considered in such study presented with any degree of ADQ muscle atrophy^(33)^.

In the present study, the authors have selected only patients with grade IV atrophy, unequivocally evidencing the presence of compressive neuropathy. Chronic plantar fasciitis and local varicosities represented the findings most frequently associated with entrapment of the abductor digiti quinti nerve. Initially, heel pain should be treated with conservative measures, including the use of a nocturnal orthosis, therapeutic footwear, physical therapy, anti-inflammatory drugs and corticoid infiltration^(16,34,35)^.

As the pain becomes chronic, over a period longer than six month and without any improvement with the conservative treatment, the hypothesis of compression of the first branch of the lateral plantar nerve should be considered. In such cases, the patients may benefit from surgical decompression of the region^(16,35-37)^ by endoscopic approach^(16,36)^, radiofrequency ablation techniques^(34)^ or open surgery.

## CONCLUSION

Atrophy of the ADQ muscle is strongly associated with neuropathic compression of the first branch of the lateral plantar nerve. MRI is considered to be a noninvasive and highly accurate diagnostic method to evaluate grade IV atrophy of ADQ muscle and other associated diseases.
